# Safety and efficacy of angiotensin-converting enzyme inhibitors in aortic stenosis: A protocol for systematic review and meta-analysis

**DOI:** 10.1097/MD.0000000000025537

**Published:** 2021-04-16

**Authors:** Chengsheng Xu, Juanjuan Xu, Juan Yang

**Affiliations:** Department of Cardiovascular Medicine, Huanggang Central Hospital, Hubei, China.

**Keywords:** angiotensin-converting enzyme inhibitors, aortic stenosis, meta-analysis, protocol

## Abstract

**Background:**

Although there are many studies showing potential benefit in aortic stenosis (AS) patients taking angiotensin-converting enzyme inhibitors (ACEI), but these studies are subject to significant selection and other biases, making the results challenging to interpret. Furthermore, the evidence on the use of ACEI in AS patients has not been reviewed systematically; we thus conducted this protocol assess the clinical effectiveness and safety of ACEI for patients with AS.

**Methods:**

The following search terms will be used in PUBMED, Scopus, EMBASE, and Cochrane Library databases on May, 2021, as the search algorithm: (angiotensin-converting enzyme inhibitors) OR (ACEI) AND (aortic stenosis) OR (AS). Two searchers will independently draft and carry out the search strategy, and the third member will further complete it. The studies on cohort study focusing on assessing the efficacy of ACEI on AS patients will be included in our meta-analysis. At least one of the following outcomes should have been measured: left ventricular mass, exercise tolerance, B-type natriuretic peptide, adverse event, functional outcomes, and aortic valve area. All outcomes are pooled on random-effect model. A *P* value of <.05 is considered to be statistically significant.

**Results:**

The results of this research will be delivered in a peer-reviewed journal.

**Conclusion:**

Depending on the previous studies, we assumed that ACEI could possibly improve the clinical symptoms and outcomes of symptomatic AS.

**Systematic review registration number:**

10.17605/OSF.IO/G9KPT.

## 1 Introduction

Aortic stenosis (AS) is a common valve disease of the heart worldwide that exposes the left ventricle to chronic pressure overload. This triggers a complex cascade of reactions leading to the processes of left ventricular remodeling, leading to hypertrophy and fibrosis.^[1]^ If treatment is carried out too late, the regression of these processes of left ventricular remodeling will be reduced and morbidity and mortality will increase.^[2]^

Animal models have demonstrated that angiotensin-converting enzyme inhibitors (ACEI) maintain ventricular mass and function in experimental aortic bundle ligation. Linopril has been shown to reduce myocardial fibrosis in systemic hypertension. The beneficial effects of ACEI are attributed only in part to vasodilation.^[3,4]^ The antiproliferative and neurohumoral effects of angiotensin II blockers are important contributions to these benefits. Long-term ACEI therapy may benefit cardiac hypertrophy and poor remodeling and alter the natural progression of left ventricular dysfunction in patients with severe AS. Various large trials have shown significant survival benefits at all stages of heart failure.^[5–7]^ Currently, ACE inhibition is the first line of treatment for the prevention and treatment of heart failure. However, ACEI has traditionally been contraindicated for moderate to severe AS due to the theoretical risk of syncope due to reduced afterload, and current guidelines still recommend caution. However, no clinical studies have shown harm, and in fact, limited animal and human data do not suggest harm, or even a benefit.^[8–10]^

Although there are many studies showing potential benefit in AS patients taking ACEI, but these studies are subject to significant selection and other biases, making the results challenging to interpret.^[6,9]^ Furthermore, the evidence on the use of ACEI in AS patients has not been reviewed systematically; we thus conducted this protocol assess the clinical effectiveness and safety of ACEI for patients with AS. Depending on the previous studies, we assumed that ACEI could possibly improve the clinical symptoms and outcomes of symptomatic AS.

## 2 Materials and methods

### 2.1 Data sources and search strategy

The systematic literature review is structured to adhere to PRISMA guidelines (Preferred Reporting Items for Systematic Reviews and Meta-analyses), which include requirements deemed essential for the transparent reporting of results. We will update our protocol for any changes in the entire research process if needed. The following search terms will be used in PUBMED, Scopus, EMBASE, and Cochrane Library databases on May, 2021, as the search algorithm: (angiotensin-converting enzyme inhibitors) OR (ACEI) AND (aortic stenosis) OR (AS). Two searchers will independently draft and carry out the search strategy, and the third member will further complete it. References within included articles are reviewed to include articles that are not included within our literature search. The systematic review protocol has been registered on Open Science Framework registries. The registration number is 10.17605/OSF.IO/G9KPT. Since this study is on the basis of published or registered studies, ethical approval and informed consent of patients are not required.

### 2.2 Eligibility criteria

The studies on cohort study focusing on assessing the efficacy of ACEI on AS patients will be included in our meta-analysis. At least one of the following outcomes should have been measured: left ventricular mass, exercise tolerance, B-type natriuretic peptide, adverse event, functional outcomes, and aortic valve area. The exclusion criteria contain biochemical trials, reviews, case reports, no assessment of outcomes mentioned above, and no assessing the efficacy of ACEI on AS patients.

### 2.3 Data extraction

Two independent authors will extract the following descriptive raw information from the selected studies: study characteristics such as author, publication year, study design; patient demographic details such as patients’ number, average age, body mass index, and sex ratio. The outcomes measures include left ventricular mass, exercise tolerance, B-type natriuretic peptide, adverse event, functional outcomes, and aortic valve area. Where disagreement in the collection of data occurs, this is resolved through discussion. If the data are missing or cannot be extracted directly, we will contact the corresponding authors to ensure that the information integrated. Otherwise, we calculate them with the guideline of Cochrane Handbook for Systematic Reviews of Interventions. If necessary, we will abandon the extraction of incomplete data.

### 2.4 Statistical analysis

Review Manager software (v 5.3; Cochrane Collaboration) is used for the meta-analysis. Extracted data are entered into Review Manager by the first independent author and checked by the second independent author. Risk ratio with a 95% confidence interval or standardized mean difference with 95% CI is assessed for dichotomous outcomes or continuous outcomes, respectively. The heterogeneity is assessed by using the Q test and *I*^2^ statistic. An *I*^2^ value of <25% is chosen to represent low heterogeneity and an *I*^2^ value of >75% to indicate high heterogeneity. All outcomes are pooled on random-effect model. A *P* value of <.05 is considered to be statistically significant.

### 2.5 Quality assessment

The Cochrane risk of bias tool is used to evaluate the risk of bias of included randomized controlled trials by 2 independent reviewers. The quality of randomized controlled trials is assessed by using following 7 items: random sequence generation, allocation concealment, blinding of participants and personnel, blinding of outcome assessment, incomplete outcome data, selective reporting, and other bias. Disagreement is resolved through discussion and consensus between the reviewers. Kappa values will be used to measure the degree of agreement between the 2 reviewers and are rated as follows: fair, 0.40 to 0.59; good, 0.60 to 0.74; and excellent, ≥0.75. Based on the information provided from included studies, each item is recorded as low risk of bias, high risk of bias, or unclear (lack of information or unknown risk of bias). We also conduct the sensitivity analysis to evaluate whether any single study have the weight to skew on the overall estimate and data. Begg funnel plot is used to assess publication bias. If publication bias exists, the Begg funnel plot is asymmetric.

## 3 Discussion

AS is the most common form of valvular heart disease in the Western world affecting 5% of those aged >75 years. Given the direct deleterious effects of renin–angiotensin system activation on the myocardium in AS, ACEI may moderate myocardial hypertrophy and fibrosis and may have a beneficial effect on left ventricular remodeling in patients with severe AS. Although there are many studies showing potential benefit in AS patients taking ACEI, but these studies are subject to significant selection and other biases, making the results challenging to interpret. Furthermore, the evidence on the use of ACEI in AS patients has not been reviewed systematically; we thus conducted this protocol assess the clinical effectiveness and safety of ACEI for patients with AS. Depending on the previous studies, we assumed that ACEI could possibly improve the clinical symptoms and outcomes of symptomatic AS.

## Author contributions

**Conceptualization:** Juan Yang.

**Data curation:** Chengsheng Xu, Juanjuan Xu, Juan Yang.

**Formal analysis:** Chengsheng Xu, Juanjuan Xu.

**Funding acquisition:** Chengsheng Xu.

**Investigation:** Chengsheng Xu, Juanjuan Xu.

**Methodology:** Chengsheng Xu, Juanjuan Xu, Juan Yang.

**Project administration:** Chengsheng Xu.

**Resources:** Chengsheng Xu.

**Software:** Juanjuan Xu, Juan Yang.

**Supervision:** Juanjuan Xu, Juan Yang.

**Validation:** Juanjuan Xu, Juan Yang.

**Visualization:** Juanjuan Xu.

**Writing – original draft:** Chengsheng Xu.

**Writing – review & editing:** Juanjuan Xu, Juan Yang.

## References

[1] Capoulade R Clavel MA Mathieu P. Impact of hypertension and renin-angiotensin system inhibitors in aortic stenosis. Eur J Clin Invest 2013;43:1262–72. 24117162 10.1111/eci.12169

[2] He S Fang Z. Incidence, predictors, and outcome of prosthesis-patient mismatch after transcatheter aortic valve replacement: a meta-analysis. Medicine (Baltimore) 2020;99:e20717. 32541522 10.1097/MD.0000000000020717 PMC7302587

[3] Chen S Redfors B Nazif T. Impact of renin-angiotensin system inhibitors on clinical outcomes in patients with severe aortic stenosis undergoing transcatheter aortic valve replacement: an analysis of from the PARTNER 2 trial and registries. Eur Heart J 2020;41:943–54. 31711153 10.1093/eurheartj/ehz769 PMC8204653

[4] Bang CN Greve AM Køber L. Renin-angiotensin system inhibition is not associated with increased sudden cardiac death, cardiovascular mortality or all-cause mortality in patients with aortic stenosis. Int J Cardiol 2014;175:492–8. 25012498 10.1016/j.ijcard.2014.06.013

[5] Chockalingam A Venkatesan S Subramaniam T. Safety and efficacy of angiotensin-converting enzyme inhibitors in symptomatic severe aortic stenosis: Symptomatic Cardiac Obstruction-Pilot Study of Enalapril in Aortic Stenosis (SCOPE-AS). Am Heart J 2004;147:E19. 15077102 10.1016/j.ahj.2003.10.017

[6] Rosenhek R Rader F Loho N. Statins but not angiotensin-converting enzyme inhibitors delay progression of aortic stenosis. Circulation 2004;110:1291–5. 15337704 10.1161/01.CIR.0000140723.15274.53

[7] Bull S Loudon M Francis JM. A prospective, double-blind, randomized controlled trial of the angiotensin-converting enzyme inhibitor Ramipril In Aortic Stenosis (RIAS trial). Eur Heart J Cardiovasc Imaging 2015;16:834–41. 25796267 10.1093/ehjci/jev043 PMC4505792

[8] Ardehali R Leeper NJ Wilson AM. The effect of angiotensin-converting enzyme inhibitors and statins on the progression of aortic sclerosis and mortality. J Heart Valve Dis 2012;21:337–43. 22808835 PMC4182019

[9] Wakabayashi K Tsujino T Naito Y. Administration of angiotensin-converting enzyme inhibitors is associated with slow progression of mild aortic stenosis in Japanese patients. Heart Vessels 2011;26:252–7. 21063877 10.1007/s00380-010-0052-x

[10] Muttardi K Haydar A Phua CK. An underused opportunity to introduce ACE inhibitors and influence prognosis: observational study of patients undergoing aortic surgery. JRSM Short Rep 2013;4: 2042533313484145. 10.1177/2042533313484145 PMC3697859 23885293

